# Gene–gene and gene-environment interactions on cord blood total IgE in Chinese Han children

**DOI:** 10.1186/s13223-021-00571-0

**Published:** 2021-07-09

**Authors:** Li Hua, Quanhua Liu, Jing Li, Xianbo Zuo, Qian Chen, Jingyang Li, Yuwei Wang, Haipei Liu, Zhaobo Shen, Yi Li, Zenan Ma, Shengdong Dong, Ruoxu Ji, Dingzhu Fang, Yi Chen, Wenwei Zhong, Jun Zhang, Jianhua Zhang, Yixiao Bao

**Affiliations:** 1 Department of Pediatric Pulmonology, Xin Hua Hospital, Shanghai Jiao Tong University School of Medicine, 1665 Kongjiang Road, Shanghai, 200092 China; 2 Ministry of Education-Shanghai Key Laboratory of Children’s Environmental Health, Xin Hua Hospital, Shanghai Jiao Tong University School of Medicine, Shanghai, China; 3 Shanghai Tonxin Pediatric Clinic, Shanghai, China; 4 Key Laboratory of Dermatology, Ministry of Education, Anhui Medical University, Hefei, Anhui China; 5 Department of Pulmonology, Children’s Hospital, Zhengzhou University, Zhengzhou, Henan China; 6 Department of Pediatrics, Suzhou Wuzhong People’s Hospital, Suzhou, Jiangsu China; 7 Medical Department, Shanghai Seventh People’s Hospital, Shanghai University of Traditional Chinese Medicine, Shanghai, China; 8 Department of Ophthalmology, Shanghai General Hospital, Shanghai Jiao Tong University School of Medicine, Shanghai, China

**Keywords:** Cord blood, IgE, Gene—gene interaction, Gene—environment interaction

## Abstract

**Background:**

*IL13, IL4, IL4RA, FCER1B* and *ADRB2* are susceptible genes of asthma and atopy. Our previous study has found gene–gene interactions on asthma between these genes in Chinese Han children. Whether the interactions begin in fetal stage, and whether these genes interact with prenatal environment to enhance cord blood IgE (CBIgE) levels and then cause subsequent allergic diseases have yet to be determined. This study aimed to determine whether there are gene–gene and gene-environment interactions on CBIgE elevation among the aforementioned five genes and prenatal environmental factors in Chinese Han population.

**Methods:**

989 cord blood samples from a Chinese birth cohort were genotyped for nine single-nucleotide polymorphisms (SNPs) in the five genes, and measured for CBIgE levels. Prenatal environmental factors were collected using a questionnaire. Gene–gene and gene-environment interactions were analyzed with generalized multifactor dimensionality methods.

**Results:**

A four-way gene–gene interaction model (*IL13* rs20541, *IL13* rs1800925, *IL4* rs2243250 and *ADRB2* rs1042713) was regarded as the optimal one for CBIgE elevation (testing balanced accuracy = 0.5805, *P* = 9.03 × 10^–4^). Among the four SNPs, only *IL13* rs20541 was identified to have an independent effect on elevated CBIgE (odds ratio (OR) = 1.36, *P* = 3.57 × 10^–3^), while the other three had small but synergistic effects. Carriers of *IL13* rs20541 TT, *IL13* rs1800925 CT/TT, *IL4* rs2243250 TT and *ADRB2* rs1042713 AA were estimated to be at more than fourfold higher risk for CBIgE elevation (OR = 4.14, *P* = 2.69 × 10^–2^). Gene-environment interaction on elevated CBIgE was found between *IL4* rs2243250 and maternal atopy (OR = 1.41, *P* = 2.65 × 10^–2^).

**Conclusions:**

Gene–gene interaction between *IL13* rs20541, *IL13* rs1800925, *IL4* rs2243250 and *ADRB2* rs1042713, and gene-environment interaction between *IL4* rs2243250 and maternal atopy begin in prenatal stage to augment IgE production in Chinese Han children.

## Introduction

The worldwide prevalence of allergic diseases has dramatically increased during the past few decades, resulting in heavy burden to the whole society and huge medical expenditure around the world [1]. Allergic diseases have long been attributed to IgE-mediated inflammatory responses [2]. Evidence has demonstrated that regulation of IgE production may begin in utero, reflected in the levels of cord blood IgE (CBIgE) [3]. Elevated CBIgE has been shown to be a risk factor for the subsequent development of allergic diseases [4]. Recent studies have indicated that certain genes and environmental factors may interact to elevate CBIgE levels [5–7], with the heritability estimated around 84–95% [8].

Ober and Hoffjan reviewed 118 genes associated with asthma or atopy, among which 25 have been replicated in six or more independent samples and thus are considered to be true susceptibility genes [9]. The elite group of susceptible genes of asthma and atopy replicated in more than ten different studies include *IL13, IL4, IL4RA, FCER1B* and *ADRB2*, five important inflammatory genes associated with IgE levels [10–12]. Our previous study has found that gene–gene interactions on childhood asthma exist between these genes in Chinese Han population [13]. Whether the gene–gene interactions among the aforementioned five genes begin in fetal stage, and whether these genes interact with prenatal environment to enhance CBIgE production and then cause subsequent allergic diseases have yet to be determined.

This study attempts to explore whether there are gene–gene and gene-environment interactions on CBIgE elevation among genetic variants in *IL13, IL4, IL4RA, FCER1B* and *ADRB2* genes and prenatal environmental factors in Chinese Han population. This is the first study to investigate gene–gene and gene-environment interactions on CBIgE in the mainland of China. Elucidation of genetic and environmental determinants of CBIgE may allow for detection and prevention of allergic sensitization in early life.

## Methods

### Study participants

This study included 989 Chinese Han children from the Shanghai Allergy Cohort, which was a prospective birth cohort with infants recruited between 2012 and 2013 at two large tertiary hospitals in Shanghai, Xinhua Hospital and the International Peace Maternity & Child Health Hospital. Written informed consent was obtained from the mothers prior to delivery. Prenatal and perinatal epidemiologic and clinical information along with cord blood samples were collected by trained research nurses. The study was approved by the Ethics Committee of Xinhua Hospital and the International Peace Maternity & Child Health Hospital (approval number: XHEC-C-2012–003), and conducted according to the principles in the Declaration of Helsinki.

### Epidemiologic and clinical information collection

Trained research nurses conducted face-to-face interviews using structured questionnaires, collecting information on maternal age, height, prepregnancy weight, education level, maternal atopy, prenatal pet exposure, prenatal active or secondhand smoking, and family income. Maternal atopy was referred to those mothers who had asthma, allergic rhinitis or atopic dermatitis along with detectable specific IgE. Prenatal pet exposure was defined as keeping cats or dogs at home during pregnancy.

Information on parity, previous pregnancy, gestational age, date of birth, delivery mode, infants’ gender, birth weight and antenatal complications was obtained from medical records.

### CBIgE measurement

CBIgE levels were determined by using ImmunoCAP Total IgE Low Range Assay [5] on the Phadia 250 (Thermo Scientific™, Waltham, Massachusetts, USA) according to the standard manufacturer’s protocols. Elevation of CBIgE levels was cut-off at ≥ 0.5 KU/L as previously described [5, 6].

### Selection of genes and single nucleotide polymorphisms

This study focused on five candidate genes, including *IL13, IL4, IL4RA, FCER1B* and *ADRB2*, which are key inflammatory genes affecting IgE levels [10–12] and had been found associated with asthma or atopy by more than ten different studies [9]. Our previous study had identified gene–gene interactions on asthma between these genes in Chinese Han children [13]. Within these genes, nine known functional single-nucleotide polymorphisms (SNPs) [13] with minor allele frequency greater than 10% were chosen for analysis, as shown in Table 1.

**Table 1:** Candidate genes and SNPs analyzed in this study

Gene	SNP	rs Number	Chromosome position	Location
*IL13*	− 1112C > T	rs1800925	5:132,657,117	Promoter
*IL13*	+ 1923C > T	rs1295686	5:132,660,151	Intron 3
*IL13*	R110Q	rs20541	5:132,660,272	Exon 4
*IL4*	− 590C > T	rs2243250	5:132,673,462	Promoter
*ADRB2*	R16G	rs1042713	5:148,826,877	Exon1
*FCER1B*	− 109C > T	rs1441586	11:60,088,555	Promoter
*FCER1B*	E237G	rs569108	11:60,095,631	Exon7
*IL4RA*	I75V	rs1805010	16:27,344,882	Exon 5
*IL4RA*	Q551R	rs1801275	16:27,363,079	Exon 12

*SNP* single-nucleotide polymorphism,* rs* reference SNP

### Genotyping

Genomic DNA was extracted from cord blood using QIAamp DNA Blood Mini Kit (QIAGEN, Hilden, Germany). Genotyping of the nine SNPs was performed by matrix-assisted laser desorption / ionization time of flight mass spectrometry (MALDI-TOF MS) [14] using the MassARRAY iPLEX platform (Sequenom Inc, San Diego, CA, USA) according to the manufacturer’s instructions. Laboratory personnel were blinded to CBIgE status. The overall call rate was 98.6%. Genotyping quality control included 5% duplicate and negative samples. Genotyping concordance rate was higher than 98%.

### Statistical analysis

Associations between CBIgE elevation and the epidemiologic characteristics of the study subjects were assessed by the χ^2^ test. The Hardy–Weinberg equilibrium test for each of the nine SNPs was performed in the total population with the χ^2^ statistics. Association of elevated CBIgE in subjects with each SNP was analyzed by using the Pearson’s χ^2^ test. In addition to the allelic test of association, dominant and recessive genetic models were tested for the nine SNPs by logistic regression analysis. *P* value, odds ratio (OR) and 95% confidence interval (95% CI) were calculated by using the PLINK program (http://pngu.mgh.harvard.edu/~purcell/plink/). A two-tailed *P* value ≤ 0.0055 after Bonferroni Multiple Testing correction was considered statistically significant.

Gene–gene interactions were analyzed with GMDR (Version 1.0), which is a free, open-source interaction analysis tool, aimed to perform gene–gene interaction with generalized multifactor dimensionality reduction (GMDR) methods [15]. The model that maximizes the testing balanced accuracy (TBA) and minimizes the statistical significance is selected. TBA indicates the accuracy of classification of cases and controls. Heuristically, a satisfactory TBA is higher than 0.55. Gene–gene interactions revealed by GMDR analyses were validated by χ^2^ tests. Gene-environment interactions were evaluated by logistic regression analysis and GMDR approach. Linkage disequilibrium (LD) was calculated for the SNPs located on one chromosome. The detection power of the sample size in this study was 0.88 based on the minor allele frequency of 0.25 and its OR for CBIgE elevation at 1.30.

## Results

### Association between CBIgE elevation and the epidemiologic characteristics of the study subjects

There were 989 Chinese Han infants in this study, of whom 27.1% had elevated CBIgE levels. Table 2 presents the distribution of CBIgE concentrations by epidemiologic characteristics of the study subjects. Cesarean section and male gender were associated with elevated CBIgE levels (*P* < 0.05).

**Table 2:** Associations between CBIgE elevation and the epidemiologic characteristics of the study subjects

Phenotypes	N (%)	Elevated rate of CBIgE	*P* value^*^
Maternal age (y)			
< 25	62 (6.4)	17 (27.4)	6.92 × 10^–1^
25–29	500 (51.9)	128 (25.6)	
30–34	325 (33.7)	92 (28.3)	
≥ 35	77 (8.0)	24 (31.2)	
Maternal prepregnancy BMI (kg/m^2^)			
< 18.5	150 (15.6)	42 (28.0)	5.79 × 10^–1^
18.5–24.9	694 (72.1)	182 (26.2)	
25–29.9	92 (9.6)	29 (31.5)	
≥ 30	26 (2.7)	9 (34.6)	
Maternal education			
Middle school or lower	28 (2.9)	6 (21.4)	5.48 × 10^–1^
High school	112 (11.6)	27 (24.1)	
College or higher	825 (85.5)	230 (27.9)	
Family income (CNY)			
< 100 K	273 (27.6)	74 (27.1)	9.98 × 10^–1^
≥ 100 K	540 (54.6)	146 (27.0)	
Unknown	176 (17.8)	48 (27.3)	
Maternal atopy^a^			
No	824 (86.5)	219 (26.6)	2.17 × 10^–1^
Yes	129 (13.5)	41 (31.8)	
Prenatal pet exposure^b^			
No	857 (89.1)	234 (27.3)	8.90 × 10^–1^
Yes	105 (10.9)	28 (26.7)	
Prenatal active or secondhand smoking			
No	577 (59.9)	158 (27.4)	9.32 × 10^–1^
Yes	387 (40.1)	105 (27.1)	
Parity			
None	868 (89.9)	235(27.1)	9.20 × 10^–1^
≥ 1	98 (10.1)	27 (27.6)	
Previous pregnancy			
None	629 (65.1)	169 (26.9)	8.08 × 10^–1^
≥ 1	337 (34.9)	93 (27.6)	
Gestational age (wk)			
< 37	33 (3.4)	7 (21.2)	
37–39	686 (71.0)	196 (28.6)	2.70 × 10^–1^
≥ 40	247 (25.6)	59 (23.9)	
Season of birth			
Summer (Jun.—Aug.)	170 (17.7)	46 (27.1)	4.90 × 10^–1^
Autumn (Sep.—Nov.)	451 (47.1)	131 (29.0)	
Winter (Dec.—Feb.)	337 (35.2)	85 (25.2)	
Delivery mode			
Vaginal	229 (23.7)	50 (21.8)	3.93 × 10^–2^
Cesarean section	737 (76.3)	212 (28.8)	
Gender			
Boy	499 (51.7)	156 (31.3)	2.28 × 10^–3^
Girl	466 (48.3)	105 (22.5)	
Birth weight (g)			
< 2500	24 (2.5)	8 (33.3)	7.86 × 10^–1^
2500–4000	857 (88.7)	231 (27.0)	
≥ 4000	85 (8.8)	23 (27.1)	
Antenatal complications^c^			
No	763 (81.2)	206 (27.0)	6.26 × 10^–1^
Yes	177 (18.8)	51 (28.8)	

The missing data: maternal age (n = 25); maternal prepregnancy BMI (n = 27); maternal education (n = 24); maternal atopy (n = 36); prenatal pet exposure (n = 27); prenatal active or secondhand smoking (n = 25); parity (n = 23); previous pregnancy (n = 23); gestational age (n = 23); season of birth (n = 31); delivery mode (n = 23); gender (n = 24); birth weight (n = 23); antenatal complications (n = 49). The missing data were not from the same individuals for each variable

*CBIgE* cord blood IgE

^a^Maternal atopy was referred to those mothers who had asthma, allergic rhinitis or atopic dermatitis along with detectable specific IgE

^b^Keeping cats or dogs at home during pregnancy

^c^Pregnancy hypertension, diabetes, infection or intrauterine growth retardation

^*^χ^2^ test was used to analyze associations between CBIgE elevation and the epidemiologic characteristics

### Association between CBIgE elevation and single SNPs

All the nine SNPs were in Hardy–Weinberg equilibrium (P > 0.05). As shown in Table 3, SNPs *IL13* rs1295686 and *IL13* rs20541 were solely associated with CBIgE elevation. The A allele of rs1295686 (OR = 1.37, *P* = 2.73 × 10^–3^) and T allele of rs20541 (OR = 1.36, *P* = 3.57 × 10^–3^) were significantly increased in elevated CBIgE group compared with normal group. The most significant association with CBIgE elevation was found under recessive model for the two SNPs. Significant association with CBIgE elevation was not found among the other seven loci (P > 0.0055, after Bonferroni Multiple Testing correction).

**Table 3:** Genetic effects of single SNPs on CBIgE elevation

SNP	CBIgE levels	Risk allele	Risk allele frequency, n (%)	*P* value ^*^OR(95%CI)	Genotype frequency AA/AB/BB n (%)	Dominant^†^ *P* value^‡^ OR(95%CI)	Recessive^†^ *P* value^‡^ OR(95%CI)
*IL13*					CC/CT/TT		
rs1800925	Elevated	T	102 (19.2)	5.17 × 10^–2^	170(63.9)/90(33.8)/6(2.3)	2.79 × 10^–2^	9.77 × 10^–1^
	Normal		223 (15.5)	1.29 (1.00–1.67)	512(71.2)/191(26.6)/16(2.2)	1.40 (1.04–1.88)	1.01 (0.39–2.62)
*IL13*					GG/GA/AA		
rs1295686	Elevated	A	204 (38.5)	2.73 × 10^–3^	97(36.6)/132(49.8)/36(13.6)	8.71 × 10^–3^	2.18 × 10^–2^
	Normal		451 (31.3)	1.37 (1.12–1.69)	331(46.0)/327(45.4)/62(8.6)	1.47 (1.10–1.97)	1.67 (1.08–2.58)
*IL13*					CC/CT/TT		
rs20541	Elevated	T	203 (38. 5)	3.57 × 10^–3^	97(36.7)/131(49.6)/36(13.6)	1.23 × 10^–2^	2.07 × 10^–2^
	Normal		453 (31.5)	1.36 (1.11–1.68)	329(45.7)/329(45.7)/62(8.6)	1.45 (1.08–1.94)	1.68 (1.08–2.60)
*IL4*					TT/TC/CC		
rs2243250	Elevated	C	109 (20.6)	7.55 × 10^–1^	168(63.4)/85(32.1)/12(4.5)	7.00 × 10^–1^	9.71 × 10^–1^
	Normal		287 (19.9)	1.04 (0.81–1.33)	466(64.7)/221(30.7)/33(4.6)	1.06 (0.79–1.42)	0.99 (0.50–1.94)
*ADRB2*					AA/AG/GG		
rs1042713	Elevated	A	296 (61. 7)	1.41 × 10^–1^	97(40.4)/102(42.5)/41(17.1)	8.81 × 10^–2^	5.94 × 10^–1^
	Normal		763 (57.8)	1.17 (0.95–1.46)	226(34.2)/311(47.1)/123(18.6)	0.77 (0.57–1.04)	0.90 (0.61–1.33)
*FCER1B*					TT/TC/CC		
rs1441586	Elevated	C	187 (35.4)	3.41 × 10^–1^	108(40.9)/125(47.3)/31(11.7)	3.82 × 10^–1^	5.10 × 10^–1^
	Normal		477 (33.1)	1.11 (0.90–1.37)	317(44.0)/329(45.7)/74(10.3)	1.14 (0.85–1.51)	1.16 (0.74–1.81)
*FCER1B*					TT/TC/CC		
rs569108	Elevated	C	91 (17.0)	7.26 × 10^–1^	184(68.7)/77(28.7)/7(2.6)	7.47 × 10^–1^	8.20 × 10^–1^
	Normal		235 (16.3)	1.05 (0.80–1.37)	502(69.7)/201(27.9)/17(2.4)	1.05 (0.78–1.42)	1.11 (0.45–2.71)
*IL4RA*					GG/GA/AA		
rs1805010	Elevated	G	274 (51. 9)	3.22 × 10^–1^	72(27.3)/130(49.2)/62(23.5)	3.84 × 10^–1^	4.59 × 10^–1^
	Normal		708 (49.4)	1.11 (0.91–1.35)	176(24.5)/356(49.7)/185(25.8)	0.87 (0.63–1.19)	0.88 (0.63–1.23)
*IL4RA*					AA/AG/GG		
rs1801275	Elevated	A	442 (83.4)	6.66 × 10^–1^	178(67.2)/86(32.5)/1 (0.4)	6.97 × 10^–1^	3.09 × 10^–2^
	Normal		1189 (82.6)	1.06 (0.81–1.38)	493(68.5)/203(28.2)/24(3.3)	1.06 (0.79–1.43)	0.11 (0.01–0.82)

*SNP* single-nucleotide polymorphism, *CBIgE* cord blood IgE, *OR* odds ratio, *CI* confidence interval

^*^P Values for Pearson’s χ^2^ tests

^†^Dominant model (AA vs AB + BB) and recessive model (AA + AB vs BB), where A is the major allele and B is the minor allele

^‡^*P* Values for logistic analyses

### Gene–gene interactions on CBIgE elevation

Gene–gene interactions on CBIgE elevation were explored among all the nine SNPs by GMDR approach. Totally, there were four models exhibiting a TBA higher than 0.55, as shown in Table 4. Based on the TBA and *P* values, significant multi-loci interactions were found in the four models (*P* < 0.05). Among them, the four-way interaction model (*IL13* rs20541, *IL13* rs1800925, *IL4* rs2243250 and *ADRB2* rs1042713) which showed the highest TBA and lowest *P* value (TBA = 0.5805, *P* = 9.03 × 10^–4^), was regarded as the optimal one. As the four SNPs that made up the optimal model are located on one chromosome, pairwise LD of them was calculated (r^2^ < 0.3), indicating a low LD between them. Interactions between the four SNPs of the optimal model were further validated by χ^2^ tests. Table 5 shows that individuals carrying *IL13* rs20541 TT, *IL13* rs1800925 CT/TT, *IL4* rs2243250 TT and *ADRB2* rs1042713 AA had a significantly higher risk of CBIgE elevation compared with those without any of the four risk genotypes (OR = 4.14, *P* = 2.69 × 10^–2^), and also greater than those with less than four risk genotypes.

**Table 4:** Summary of gene–gene interactions for CBIgE elevation by GMDR analysis

Interacting SNPs	TBA	*P* value
*IL13* rs20541 × *ADRB2* rs1042713	0.5621	1.07 × 10^–2^
*IL13* rs1800925 × *IL4* rs2243250 × *ADRB2* rs1042713	0.5591	7.77 × 10^–3^
*IL13* rs20541 × *IL13* rs1800925 × *IL4* rs2243250 × *ADRB2* rs1042713	0.5805	9.03 × 10^–4^
*IL13* rs20541 × *IL13* rs1800925 × *IL13* rs1295686 × *IL4* rs2243250 × *ADRB2* rs1042713	0.5724	1.53 × 10^–3^

*CBIgE* cord blood IgE, *GMDR* generalized multifactor dimensionality reduction, *SNP* single-nucleotide polymorphism, *TBA* Testing balanced accuracy

**Table 5:** Interactions between *IL13* rs20541, *IL13* rs1800925, *IL4* rs2243250 and *ADRB2* rs1042713 genotypes for CBIgE elevation

Number of risk genotype for the four SNPs ^a^	CBIgE levels		*P *value ^†^	OR (95%CI)
	Elevated, n (%)	Normal, n (%)		
0	31 (13.2)	110 (16.8)		1
1	92 (39.1)	265 (40.4)	3.78 × 10^–1^	1.23 (0.78–1.96)
2	71 (30.2)	217 (33.1)	5.42 × 10^−1^	1.16 (0.72–1.88)
3	34 (14.5)	58 (8.8)	1.28 × 10^–2^	2.08 (1.16–3.72)
4	7 (3.0)	6 (0.9)	2.69 × 10^–2^	4.14 (1.30–13.22)

*CBIgE* cord blood IgE, *SNP* single-nucleotide polymorphism, *OR* odds ratio, *CI* confidence interval

^a^Risk genotypes were TT, CT/TT, TT, and AA for rs20541, rs1800925, rs2243250, and rs1042713, respectively

^†^*P* Values for χ^2^ tests

### Gene-environment interactions on CBIgE elevation

Logistic regression analysis and GMDR approach were applied to search the potential gene-environment interactions on CBIgE elevation between the nine SNPs and environmental factors including prenatal pet exposure, prenatal active or secondhand smoking, maternal atopy, maternal age, maternal prepregnancy BMI, delivery mode, infants’ gender and season of birth. By using logistic regression analysis, it was found that C allele of *IL4* rs2243250 interacted with maternal atopy to elevate CBIgE levels (OR = 1.41, *P* = 2.65 × 10^–2^), as shown in Table 6. However, no significant gene-environment interaction was found by GMDR analysis.

**Table 6:** Interactions between the nine SNPs and maternal atopy for CBIgE elevation

Gene	SNP	Minor allele	OR(95%CI)	*P* value^*^
*IL13*	rs1800925	T	1.21 (0.86–1.71)	2.81 × 10^–1^
*IL13*	rs1295686	A	0.89 (0.67–1.20)	4.53 × 10^–1^
*IL13*	rs20541	T	0.91 (0.68–1.22)	5.25 × 10^–1^
*IL4*	rs2243250	C	1.41 (1.04–1.91)	2.65 × 10^–2^
*ADRB2*	rs1042713	G	0.86 (0.66–1.14)	2.94 × 10^–1^
*FCER1B*	rs1441586	C	1.21 (0.91–1.59)	1.91 × 10^–1^
*FCER1B*	rs569108	C	1.19 (0.84–1.69)	3.19 × 10^–1^
*IL4RA*	rs1805010	A	0.89 (0.68–1.16)	3.77 × 10^–1^
*IL4RA*	rs1801275	G	1.23 (0.88–1.72)	2.31 × 10^–1^

*SNP* single-nucleotide polymorphism, *CBIgE* cord blood IgE, *OR* odds ratio, *CI* confidence interval

^*^P Values were tested by multivariate logistic regression, adjusted for other genes, but not for other environmental factors

## Discussion

IgE-mediated reaction is the central component of allergic diseases. Five key inflammatory genes affecting IgE levels, including *IL13, IL4, IL4RA, FCER1B* and *ADRB*2 [10–12], have been demonstrated associated with asthma or atopy by more than ten different studies [9]. Our previous study has found that gene–gene interactions on asthma exist between these genes in Chinese Han children [13]. This study attempted to determine whether the interactions begin in utero, and whether these genes interact with prenatal environmental factors to increase CBIgE levels and induce subsequent allergic diseases. Of the models tested using GMDR approach, the four-way gene–gene interaction model consisting of *IL13* rs20541, *IL13* rs1800925, *IL4* rs2243250 and *ADRB2* rs1042713 was chosen as the optimal one for CBIgE elevation based on its TBA and *P* value. Among the four SNPs, only *IL13* rs20541 was identified to have an independent effect on CBIgE elevation, while the other three had small but synergistic effects. Carriers of *IL13* rs20541 TT, *IL13* rs1800925 CT/TT, *IL4* rs2243250 TT and *ADRB2* rs1042713 AA were estimated to be at more than fourfold higher risk for CBIgE elevation. Among these genes and prenatal environmental factors, only *IL4* rs2243250 and maternal atopy were found to have interactions on elevated CBIgE. This is the first study to elucidate genetic and environmental determinants of CBIgE in Han population of mainland China.

To our knowledge, this study is also the first to identify gene–gene interactions between *IL13* rs20541, *IL13* rs1800925, *IL4* rs2243250 and *ADRB2* rs1042713 on CBIgE elevation. *IL13* and *IL4* genes encode cytokines interleukin 13 (IL13) and IL4, which share a common signaling pathway in binding to their receptors on human B cells, and switch immunoglobulin production from IgM to IgE [16]. *ADRB2* gene encodes Beta2-adrenergic receptor (ADRB2). Stimulation of ADRB2 on B cells responding to allergen enhances IgE production via a unique signaling pathway, independently of class switch recombination [17, 18]. IL13, IL4 and ADRB2 are all associated with IgE levels. *IL13* rs20541 TT genotype, *IL13* rs1800925 T allele, *IL4* rs2243250 TT genotype and *ADRB2* rs1042713 AA genotype have been associated with increased IL13 concentration [19], enhanced *IL13* promoter activity [20], augmented IL4 levels [21], and decreased downregulation of ADRB2 [22], respectively. How these four varients interact with each other biologically to promote IgE production in prenatal stage need further functional studies in vitro and in vivo.

In this study, gene-environment interaction on elevated CBIgE was found between *IL4* rs2243250 and maternal atopy. Maternal atopy has been reported to modify cord blood immune response and it may provide an intrauterine environment that influences fetal immune development and results in allergic predisposition [23–25]. *IL4* gene polymorphism affects cytokine IL4 levels [26]. How maternal atopy interacts with *IL4* gene variants to enhance antenatal IgE production need future biological studies.

Our study confirmed the independent role of *IL13* rs20541 and rs1295686 on CBIgE elevation, and also found the association of cesarean section and male gender with elevated CBIgE levels, consistent with previous reports [3, 5–7, 27]. However, no interactions were identified among them. To date, only a few studies have explored gene–gene and gene-environment interactions on CBIgE elevation. One study in a predominantly black sample reported that three *IL13* SNPs (rs1295686, rs1800925 and rs206974) could jointly influence CBIgE concentration [3]. One study in a birth cohort in Korea identified interactions between reactive oxygen species genes, prenatal exposure to home renovation and maternal atopy on CBIgE response [28]. Another study, in a Chinese population in Taiwan, found that *IL13* rs20541, male sex and prenatal environmental tobacco smoke interacted on antenatal IgE production [5]. In this study, we found a four-way genetic interactions among *IL13* rs20541, *IL13* rs1800925, *IL4* rs2243250 and *ADRB2* rs1042713, and a two-way gene-environment interactions between *IL4* rs2243250 and maternal atopy on CBIgE elevation. The variation of the gene–gene and gene-environment interactions on fetal IgE production may be in part explained by different populations and different genetic and environmental factors focused in different studies. Therefore, when we move forward to identify constellations of interacting genes and environments that influence antenatal IgE production, replication studies in different populations are required.

There are some limitations in this study. First, only five genes (*IL13, IL4, IL4RA, FCER1B* and *ADRB2*) were chosen as candidate genes. However, these five genes are susceptible genes of asthma and atopy replicated in more than ten different studies [9], and our previous study has found that gene–gene interactions on asthma exist between these genes in Chinese Han children [13]. Second, the subjects’ environmental exposures were evaluated using a self-reported questionnaire, which might lead to an underestimation of the associations of certain environmental exposures. Genes and environmental factors interact to elevate CBIgE concentrations [5–7], with the heritability estimated around 84–95% [8]. In our future studies, more candidate genes especially those from genome-wide association studies should be included and direct measurement of certain environmental exposures is needed. Third, cord blood IgA concentrations were not measured to exclude subjects whose circulation was contaminated by maternal blood. However, previous studies using cord blood IgA levels as an indicator of maternal contamination have reported a very low rate of contamination [29]. Therefore CBIgE is unlikely to be contaminated by maternal IgE [3].

In summary, Gene–gene interaction between *IL13* rs20541, *IL13* rs1800925, *IL4* rs2243250 and *ADRB2* rs1042713, and gene-environment interaction between *IL4* rs2243250 and maternal atopy begin in fetal stage to increase IgE production in Chinese Han children. After future functional and replication studies, these findings may be translated into specific strategies for early prediction and prevention of allergy.

## Data Availability

The data that support the findings of this study are not publicly available due to ethical concerns, but are available from the corresponding authors upon reasonable request.
